# Anticoagulação Oral com AVKs: Qualidade Acima de Tudo!

**DOI:** 10.36660/abc.20240795

**Published:** 2025-03-06

**Authors:** Letícia Braga Ferreira, André Assis Lopes do Carmo

**Affiliations:** 1 Unidade de Cardiologia e Cirurgia Cardiovascular Hospital das Clínicas Universidade Federal de Minas Gerais Belo Horizonte MG Brasil Unidade de Cardiologia e Cirurgia Cardiovascular do Hospital das Clínicas da Universidade Federal de Minas Gerais, Belo Horizonte, MG – Brasil; 2 Serviço de Cardiologia e Cirurgia Cardiovascular Rede Mater Dei de Saúde Belo Horizonte MG Brasil Serviço de Cardiologia e Cirurgia Cardiovascular, Rede Mater Dei de Saúde, Belo Horizonte, MG – Brasil

**Keywords:** Fibrilação Atrial, Antagonistas de Vitamina K, Varfarina, DOAC

Desde a sua primeira aprovação pela *Food and Drug Administration* em 2010, os anticoagulantes orais diretos (DOACs) têm crescido em uso em todo o mundo e se tornaram o medicamento anticoagulante oral mais comumente prescrito inicialmente para pacientes com fibrilação atrial recentemente diagnosticada.^1^ No entanto, os altos custos ainda dificultam sua utilização, especialmente em países de baixa e média renda. Além disso, em certas condições, especialmente próteses valvares cardíacas mecânicas e estenose mitral reumática, os antagonistas da vitamina K (AVKs) continuam sendo os únicos medicamentos com segurança e eficácia estabelecidas.^2 , 3^

Dados de mais de 400.000 pacientes dos Estados Unidos mostram que, após 2019, quase 48% dos pacientes com fibrilação atrial receberam prescrição de DOAC, e apenas 17,7% estavam usando varfarina.^1^ Faltam dados tão precisos sobre a utilização de varfarina e DOAC no Brasil, mas se estima que uma proporção maior de pacientes esteja usando AVKs.^4^

Esta edição do ABC Cardiol apresenta um artigo que examina uma coorte substancial de pacientes com fibrilação atrial (FA) submetidos à terapia de anticoagulação com AVKs, com foco na incidência e nos preditores de eventos isquêmicos e hemorrágicos desfavoráveis nessa população.^5^ A incidência anual do desfecho composto de eventos tromboembólicos e morte cardiovascular foi de 4,4%, e a incidência anual de sangramentos sérios foi de 3,24% após um acompanhamento médio de 17 meses. Eventos tromboembólicos anteriores, tempo na faixa terapêutica (TTR) abaixo de 50% e menor taxa de filtração glomerular foram preditores independentes do desfecho composto, e sangramento prévio e prótese valvar mecânica foram preditores independentes de eventos hemorrágicos graves.

Os autores encontraram uma incidência impressionantemente baixa de eventos hemorrágicos. Isso pode ser parcialmente explicado pela boa qualidade geral da anticoagulação da coorte, refletida por um TTR mediano de 65%, 10%, maior do que o do estudo ROCKET-AF, por exemplo.^6 , 7^ No entanto, também é preciso estar atento às definições de desfechos ao comparar diferentes estudos. O desfecho composto de sangramento foi avaliado usando as definições da Sociedade Internacional de Trombose e Hemostasia de sangramento maior e sangramento não maior clinicamente relevante. No entanto, sangramento não maior clinicamente relevante é intrinsecamente subjetivo, e uma revisão encontrou relatos inconsistentes desse desfecho em diferentes ensaios.^8^ No ensaio ROCKET-AF,^6^ por exemplo, sangramento gengival que ocorreu espontaneamente ou durou mais de 5 minutos seria considerado sangramento não maior clinicamente relevante, mesmo que não tenha levado o paciente a procurar atendimento médico. Isso não seria mais considerado sangramento não maior clinicamente relevante após a publicação da comunicação do Comitê Científico e de Padronização da Sociedade Internacional de Trombose e Hemostasia,^8^ que definiu sangramento não maior clinicamente relevante como apenas eventos hemorrágicos que exigiram intervenção médica por um profissional de saúde, resultaram em hospitalização ou levaram a uma avaliação presencial (em oposição a apenas uma comunicação telefônica ou eletrônica). Além disso, a decisão de buscar uma avaliação clínica presencial é inerentemente subjetiva, influenciada por fatores como acessibilidade ao sistema de saúde, educação em saúde e experiências pessoais anteriores. Portanto, pelo menos algum grau da disparidade observada na incidência desse desfecho entre a coorte de Liporace e estudos randomizados anteriores pode ser atribuído às diferentes definições empregadas.

Estudos anteriores mostraram uma forte associação entre TTR e desfechos clínicos em pacientes anticoagulados com AVK. TTRs mais baixos se correlacionam com resultados mais desfavoráveis de forma quase linear, embora um limite crítico pareça estar entre 60 e 70%. TTR abaixo de 60% foi associado a uma incidência duas vezes maior de acidente vascular cerebral isquêmico ou embolia sistêmica, sangramento grave e morte por todas as causas em uma grande coorte de pacientes designados para varfarina de dois ensaios randomizados de DOAC versus AVK.^9^ Outra publicação endossando essas descobertas é uma análise *post hoc* de um ensaio randomizado comparando o tratamento com aspirina mais clopidogrel à anticoagulação com um AVK em pacientes com FA. Embora a análise principal de toda a coorte tenha demonstrado que a anticoagulação foi superior à dupla antiagregação plaquetária na prevenção de acidente vascular cerebral, infarto do miocárdio, embolia sistêmica ou morte vascular, o benefício da terapia de anticoagulação desapareceu no grupo com menor TTR quando a população foi dividida em dois subgrupos com base no TTR (abaixo e acima de 65%).^10^ Além disso, uma metanálise demonstrou que apenas um TTR acima de 70% pode tornar os AVKs igualmente seguros e eficazes como os DOACs.^11^

Uma vez que os outros preditores de eventos adversos encontrados pelos autores do estudo^5^ não são modificáveis, devemos nos concentrar em aumentar o TTR de pacientes sob terapia com AVK.^5^ Isso pode ser alcançado por meio da implementação de clínicas dedicadas à anticoagulação^12^ e do emprego de estratégias de telessaúde,^13^ ambos os quais demonstraram melhorar a qualidade da anticoagulação. Esses resultados também podem ajudar a informar a decisão sobre quem deve mudar da terapia com AVK para DOAC. Uma vez que o sistema de saúde pública brasileiro não é capaz de garantir DOAC para todos os pacientes com FA, identificar pacientes com maior risco de eventos tromboembólicos ou hemorrágicos pode ser útil na escolha daqueles que mais se beneficiariam do DOAC.

Em breve, novos estudos de custo-efetividade devem ser realizados em vista da recente expiração das patentes de DOACs no Brasil, o que reduz o preço desses medicamentos. Enquanto isso, quando se trata de anticoagulação com AVK, a qualidade da anticoagulação deve continuar sendo nossa principal meta!
