# Supplementary material for: Anticoagulação Oral com AVKs: Qualidade Acima de Tudo!
Source: Arq Bras Cardiol. 2025 Mar 6;122(2):e20240795. [Article in Portuguese] doi: 10.36660/abc.20240795 (PMC12080706; doi:10.36660/abc.20240795)
Supplement: Translation

# Translation: Oral Anticoagulation with VKAs: Quality Above All!

**DOI:** 10.36660/abc.20240795i

**Authors:** Letícia Braga Ferreira, André Assis Lopes do Carmo

**Affiliations:** 1 Brazil Unidade de Cardiologia e Cirurgia Cardiovascular do Hospital das Clínicas da Universidade Federal de Minas Gerais, Belo Horizonte, MG – Brazil; 2 Brazil Serviço de Cardiologia e Cirurgia Cardiovascular, Rede Mater Dei de Saúde, Belo Horizonte, MG – Brazil

**Keywords:** Atrial Fibrillation, Vitamin K Antagonists, Warfarin, DOAC

Since their first Food and Drug Administration approval in 2010, direct oral anticoagulants (DOACs) have grown in use worldwide and have become the most common initially prescribed oral anticoagulation drug for patients with newly diagnosed atrial fibrillation.^1^However, the high costs still hinder their utilization, especially in low and middle-income countries. Furthermore, in certain conditions, especially mechanical heart valves and rheumatic mitral stenosis, vitamin K antagonists (VKAs) remain the only drugs with established safety and efficacy.^2 , 3^

Data from more than 400.000 patients from the United States show that after 2019, nearly 48% of patients with atrial fibrillation were prescribed DOAC, and only 17,7% were using warfarin.^1^Such precise data about warfarin and DOAC utilization in Brazil are lacking but it is estimated that a higher proportion of patients are using VKAs.^4^

This edition of ABC Cardiol features an article examining a substantial cohort of atrial fibrillation (AF) patients undergoing anticoagulation therapy with VKAs, focusing on the incidence and predictors of unfavorable ischemic and hemorrhagic events within this population.^5^The annual incidence of the composite outcome of thromboembolic events and cardiovascular death was 4.4%, and the annual incidence of serious bleeding was 3.24% after a median follow-up of 17 months. Previous thromboembolic events, time in therapeutic range (TTR) below 50%, and lower glomerular filtration rate were independent predictors of the composite outcome, and previous bleeding and mechanical valve prosthesis were independent predictors of serious bleeding events.

The authors found an impressively low incidence of hemorrhagic events. This could be partially explained by the cohort’s overall good quality of anticoagulation, reflected by a median TTR of 65%, 10% greater than that of the ROCKET-AF study, for example.^6 , 7^Nevertheless, one must also be attentive to the definitions of outcomes when comparing different studies. The composite bleeding outcome was evaluated using the International Society on Thrombosis and Haemostasis definitions of major bleeding and clinically relevant non-major bleeding. However, “clinically relevant non-major bleeding” is intrinsically subjective, and a review has found inconsistent reporting of this outcome across different trials.^8^In the ROCKET-AF^6^trial, for example, gingival bleeding that occurred spontaneously or lasted for more than 5 minutes would be considered clinically relevant non-major bleeding even if it did not lead the patient to seek medical attention. This would no longer be regarded as “clinically relevant non-major bleeding” following the release of the Scientific and Standardization Committee of the International Society on Thrombosis and Hemostasis Communication,^8^which defined “clinically relevant non-major bleeding” as only hemorrhagic events that required medical intervention by a healthcare professional, resulted in hospitalization or an increased level of care, or prompted a face-to-face (as opposed to merely a telephone or electronic communication) evaluation. Furthermore, the decision to pursue an in-person clinical evaluation is inherently subjective, influenced by factors such as accessibility to the healthcare system, health literacy, and previous personal experiences. Therefore, at least some degree of the disparity observed in the incidence of this outcome between Liporace’s cohort and prior randomized studies may be attributable to the varying definitions employed.

Previous studies have shown a strong association of TTR and outcomes in patients anticoagulated with VKA. Lower TTRs correlate with poorer outcomes in a nearly linear fashion, although a critical threshold appears to be between 60 and 70%.TTR below 60% was associated with a two-fold incidence of ischemic stroke or systemic embolism, severe bleeding, and all-cause death in a large cohort of patients assigned to warfarin from two randomized trials of DOAC versus VKA.^9^Another publication endorsing these findings is a *post-hoc* analysis of a randomized trial comparing treatment with aspirin plus clopidogrel to anticoagulation with a VKA in patients with AF. Although the main results for the entire cohort demonstrated that anticoagulation was superior to double antiplatelet therapy at preventing stroke, myocardial infarction, systemic embolism, or vascular death, the benefit of anticoagulation therapy disappeared in the group with lower TTR when the population was divided into two subgroups based on TTR (below and above 65%).^10^Additionally, a meta-analysis has found that only a TTR above 70% can make VKAs equally safe and effective as DOACS.^11^

Since the other predictors of adverse events found by the authors of study^5^are non-modifiable, we should focus on increasing the TTR of patients under VKA therapy.^5^This can be achieved through the implementation of dedicated anticoagulation clinics^12^and the employment of telehealth strategies,^13^both of which have been shown to improve anticoagulation quality. These results may also help inform the decision of whom to switch from VKA to DOAC therapy. If the Brazilian public health system is unable to ensure DOAC for all AF patients, identifying patients at higher risk of thromboembolic or bleeding events could be useful in choosing those who would most benefit from DOAC.

Shortly, new cost-effective studies are expected to be carried out in light of the recent expiration of DOAC patents in Brazil, which lowers the price of those medications. Meanwhile, when it comes to anticoagulation with VKA, the quality of anticoagulation should remain our main goal!
